# Spatial Segregation and Trophic Niche Divergence in Two Disjunct Populations of Wedge‐Tailed Shearwater *Ardenna pacifica* in Eastern Australia

**DOI:** 10.1002/ece3.73158

**Published:** 2026-03-12

**Authors:** Penny E. Beaver, Nicholas Carlile, Michael D. Sumner, Mary‐Anne Lea

**Affiliations:** ^1^ Institute for Marine and Antarctic Studies University of Tasmania Battery Point Tasmania Australia; ^2^ Æstrelata Restorations Kangaroo Valley New South Wales Australia; ^3^ Australian Antarctic Division Kingston Tasmania Australia

**Keywords:** *Ardenna pacifica*, diet analysis, seabird tracking, spatial segregation, stable isotope analysis, trophic niche

## Abstract

Wedge‐tailed shearwaters (*Ardenna pacifica*) are widely distributed across tropical and subtropical oceans, with their breeding range recently extending south. For populations at their southernmost breeding range, habitat use, spatial segregation, and trophic niche dynamics remain poorly understood. Here, we investigated the habitat use, spatial segregation, and trophic niche differentiation in two disjunct populations of wedge‐tailed shearwaters in eastern Australia, between 2015 and 2019. Both populations exhibited consistent spatial segregation across all years of the study. Individuals from the temperate population consistently foraged in waters off southeastern Australia. Prior to winter migration to the western Pacific Ocean (Philippine Sea), most individuals undertook a pre‐staging detour towards the subtropical frontal zone. In contrast, subtropical conspecifics exploited waters further east and north, with a small proportion undertaking a pre‐staging detour only in the first year. Stable isotope analysis of chick feathers (δ^15^N and δ^13^C) revealed trophic and habitat segregation between colonies. With the subtropical population consistently occupying a smaller trophic niche area and exhibiting lower interannual variation across all years. Both populations exhibited a high degree of interannual variability in foraging strategies and trophic niches, indicating a capacity for behavioural adaptivity in response to prey availability and oceanic conditions. This behavoural flexibility may facilitate future range expansion into more southern temperate habitats, which is important given projected climate‐driven changes to ocean dynamics in southeastern Australia.

## Graphical abstract

This study investigated habitat use and trophic niche differentiation in two wedge‐tailed shearwater populations in eastern Australia (2015 to 2019). Temperate birds foraged mainly in southeastern Australian waters and undertook a pre‐staging detour towards the subtropical frontal zone before migration, whereas subtropical birds foraged further east and north. Stable isotope analysis revealed clear trophic segregation between colonies and high interannual variability in niche use, indicating behavoural flexibility in response to oceanic conditions. Such adaptability may facilitate future range shifts under projected climate‐driven changes in ocean dynamics.

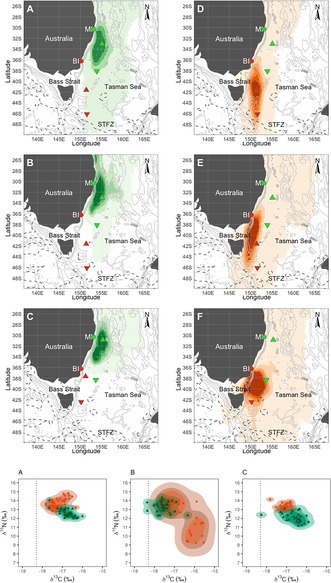

## 1 Introduction

The behavioural ecology of mesopredators such as seabirds is strongly influenced by oceanographic conditions, which influences prey availability and quality and drive behavioural adjustments in foraging strategies (Peck and Congdon 2005). Local and large‐scale environmental variability can alter prey resources, requiring seabirds to modify their behaviour to meet the energetic demands of provisioning offspring during the breeding phase (Berlincourt and Arnould 2015; Peck et al. 2004; Smithers et al. 2003).

On Australia's east coast, oceanographic conditions in the Tasman Sea are dominated by the East Australian Current (EAC). The EAC shifts warm, nutrient‐poor subtropical water southwards via mesoscale eddies that form the EAC extension (Phillips et al. 2020), before entering the eastern Bass Strait (Ridgway and Ling 2023). Climate projections indicate that the EAC will intensify and warm, with implications for marine ecosystems and prey availability for seabirds using these regions. The intensification has the potential to influence species to switch to other prey types or shift to southern latitudes where resources are more reliably consistent (Cetina‐Heredia et al. 2014; Gervais et al. 2021; Phillips et al. 2020). The strengthening of the EAC is spatially heterogeneous, with its extension along the southeastern coastline, projected to increase by ~40% and warm more rapidly than the global average (Phillips et al. 2020). Associated changes include a approximate 1.8°C increase in sea surface temperature, warming extending to depths of ~100 m, and a seasonal cycle that begins one month earlier and ends one month later (Phillips et al. 2020).

Seabirds that migrate along pathways influenced by the EAC are therefore likely to be affected by its increasing strength and variability, with some species already showing evidence of southward range expansion (Ridgeway and Hill 2009; Suthers et al. 2011). Tropical and subtropical habitats typically have lower primary productivity than temperate habitats, impacting prey availability and reliability, which can affect prey availability and put pressure on intra‐ and interspecific species (Catry et al. 2009; McDuie et al. 2015; Ravache et al. 2020; Schultz and Klomp 2000). In response to such variability, species may adapt by switching to other prey types or shift to previously unused habitats in response to oceanic changes (Phillips et al. 2020), including southward shifts where resources are generally more abundant and predictable (McDuie et al. 2015; Miller 2018; Ravache et al. 2020; Schultz and Klomp 2000). Populations competing for similar prey resources may be forced to partition their ecological niche by becoming specialists or generalists, shifting to new habitats or ocean fronts, or by having to travel farther to reach more abundant and higher quality prey resources (Corman et al. 2016). Species with generalist diets are less likely to be sensitive to changes in their environment, which enables them to be more resilient and adaptable (Polito et al. 2015).

Methods used to investigate ecological niches have expanded to include bulk stable isotopic analysis (SIA), which is now widely applied in marine ecology (Quillfeldt et al. 2005). Stable isotope ratios obtained from animal tissue, such as feathers, provide reliable biomarkers of trophic position by measuring carbon (δ^13^C) and nitrogen (δ^15^N) (Newsome et al. 2007). Carbon isotope values typically vary with marine habitat, with enrichment occurring in prey associated with inshore waters and lower latitudes compare with offshore or higher‐latitude marine habitats, particularly the Southern Ocean (Bond and Jones 2009; Hobson 1999). Nitrogen isotope values will vary according to prey ingested, with higher concentrations indicating a higher trophic level (Bond and Jones 2009). Higher latitude located in the Southern Ocean are known to have lower levels of δ^13^C and δ^15^N values due to lower iron levels, which restrict the growth of phytoplankton compared to more temperate regions (Hobson 2007).

In addition to bulk isotopic analysis, direct dietary samples using prey remains can provide complementary insights into diet composition in the short term. Such samples reflect dietary intake over periods of days to weeks, however, this method is subject to biases. Hard prey items, such as squid beaks and fish otoliths, are more likely to be preserved and remain intact, whereas soft‐bodied prey, such as euphausiids and crustaceans, are digested more rapidly and can be underrepresented (Barrett et al. 2007; Buckland et al. 2017; McCormack et al. 2019).

The wedge‐tailed shearwater (*Ardenna pacifica*) is a central place forager with a large wingspan (97–105 cm) relative to its body size (310–510 g), allowing efficient flight and foraging in light wind conditions (Spear et al. 2007). The species is widely distributed across tropical and subtropical habitats but also breeds at temperate latitudes, with numerous breeding colonies distributed along the eastern and western Australian coastlines (Marchant and Higgins 1990). Within New South Wales, 47 offshore islands provide breeding habitat for threatened, migratory, and endemic seabirds, including the wedge‐tailed shearwater. Despite their ecological importance, several temperate colonies remain poorly studied, compared with populations in tropical and subtropical regions (Cory et al. 2011; Komura et al. 2018; McDuie et al. 2015; Miller 2018; Ravache et al. 2020).

This study investigated population‐level differences in (i) spatial and temporal use; (ii) spatial segregation; (iii) trophic niche and dietary differentiation; and (iv) population dynamics, we sampled individuals from temperate and subtropical colonies of wedge‐tailed shearwaters during the breeding phase.

## 2 Materials and Methods

### 2.1 Field Methods

#### 2.1.1 Study Sites

**FIGURE 1:**
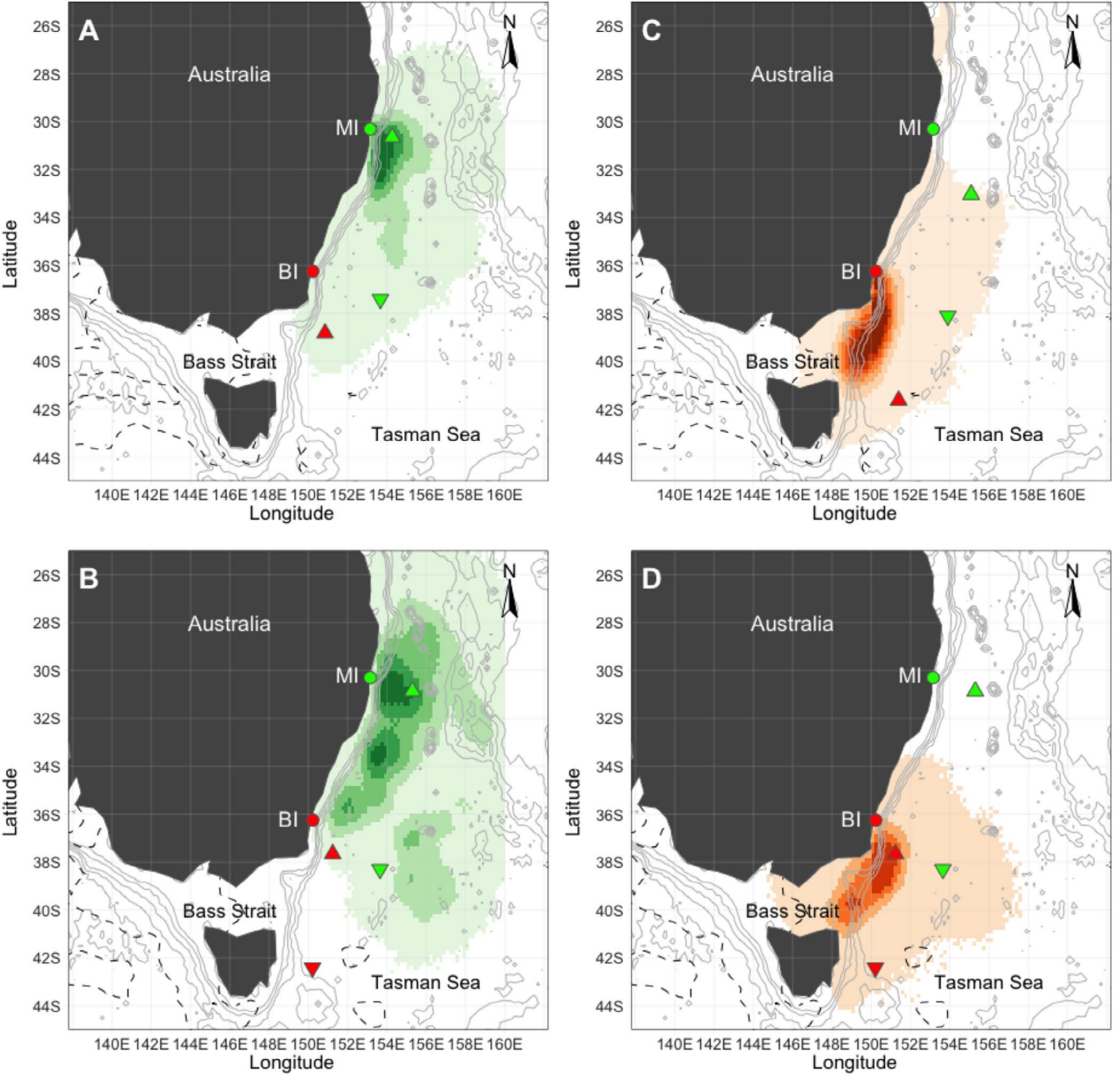
At‐sea distribution and spatiotemporal segregation of wedge‐tailed shearwaters during incubation from Muttonbird Island (MI) and Barunguba (BI). (A–B) MI 2017–2018 (*n* = 4, 6); (C–D), BI 2017–2018 (*n* = 7, 22). Green (MI) and red (BI) circles denote colony locations. North‐pointing triangles indicate the mean northern maximum location; south‐pointing triangles indicate the absolute mean southern maximum location. The dashed black line represents the approximate position of the subtropical frontal zone.

Fieldwork was conducted on Muttonbird Island (30.30° S, 153.15° E) and Barunguba, also known as Montague Island (36.25° S, 150.23° E) (Figure 1). Muttonbird Island covers nine hectares with an estimated 12,400 ± 6127 breeding pairs (Floyd and Swanson 1982), with no other seabirds breeding on the reserve during this study. Barunguba has an estimated ~47,000 breeding pairs (Peter Fullagar pers. comm., 2023) across 82 ha, comprising three shearwater species: wedge‐tailed shearwater (*A. pacifica*), short‐tailed shearwater ( *A. tenuirostris* ) and sooty shearwater ( *A. grisea* ). The island also supports several other breeding seabirds, including crested tern (*Thalasseus bergii)*, Gould's petrel (*Pterodroma leucoptera*), little penguin ( *Eudyptula minor* ), silver gull (*Larus novaehollandiae)* and white‐faced storm petrel ( *Pelagodroma marina* ) (Carlile et al. 2020).

#### 2.1.2 Tracking Device Deployment and Retrieval

Movements of shearwaters were estimated using global light sensors (GLS) attached to adult birds during the incubation and chick‐rearing phases between 2015 and 2019. Three types of devices were used: Migrate Technology Intigeo‐C65 and C65 super (weighing 2 g, including the attachment, equivalent to 0.5% of body weight) and Intigeo‐C330 (4 g, 1% of body weight). Devices were calibrated for 3–7 days prior to deployment as recommended by Lisovski et al. (2019). Ambient light levels were sampled every minute, with the maximum light level recorded every 5 min. Adults were captured by hand while occupying their burrow, and a GLS device was attached to the right tarsus using a Velcro band (C65 and C65Super, 65 mm × 10 mm, and C330, 70 mm × 14 mm). Measurements, weight, and an individual numbered steel band were attached to their left tarsus (McDuie and Congdon 2016).

During the first deployment year, all devices failed prematurely due to a manufacturing defect. Of the 139 devices deployed, 24 detached beyond the breeding colony and were not recovered, while 76 yielded usable data (55%) after being retrieved during the incubation and chick‐rearing phases. These included deployments lasting less than a year (*n* = 35), two (*n* = 30), or three (*n* = 11) years, resulting in a total of 47 individual birds included in the final analysis.

At Muttonbird Island, 19 individuals were included from devices deployed between 2015 and 2018, with three individuals providing data for 2 years. At Barunguba, 28 individuals were included from deployments between 2016 and 2019, with five individuals contributing 2 years of data and six capturing 3 years of data. The average duration of successful data recording was four and a half months in the first year (range: 2–7 months) and greater than 8 months in subsequent years (range: 8–23 months). Data was extracted using Migrate Technology Intigeo‐IF Software (v1.7, December 2015).

#### 2.1.3 Animal Tissue Collection and Processing

Body feathers were collected from both populations, sampled from randomly chosen chicks during the chick‐provisioning phase of each breeding year (2016, *n* = 77; 2017, *n* = 60; and 2018, *n* = 57). Between four and six feathers were plucked from the chest or dorsal area behind the wings (Cherel et al. 2000; Thompson et al. 2015). Large contaminants were removed by rinsing feathers with deionised water and placing them on an orbital shaker for 24–48 h. Feathers were then oven dried at 50°C for 24–48 h. Surface lipids were removed using a 2:1 solution of chloroform and methanol, after which feathers were air dried, placed in glass vials, and ground into a fine powder (Bontempo et al. 2014) for analysis at the Environmental Analysis Laboratory (Southern Cross University, Lismore, Australia).

Stable isotope ratios of carbon (δ^13^C) and nitrogen (δ^15^N) were measured using a Thermo Fisher Delta V plus isotope ratio mass spectrometer (IRMS) coupled to an elemental analyser (Thermo Fisher Flash EA) via an interface (Thermo Fisher Conflo IV). Results are expressed in parts per thousand (‰) in the typical δ notation relative to Vienna PeeDee belemnite and atmospheric N_2_ for δ^13^C and δ^15^N, respectively. Internal laboratory standards were reproduced against absolute International Atomic Energy Agency standards with a precision of 0.15‰ and 0.30‰ for δ^13^C and δ^15^N, respectively.

#### 2.1.4 Diet Sample Collection and Processing

Between January and March 2016, stomach contents were collected at both sites from randomly selected adults returning to feed chicks. Samples were obtained by lavaging the proventriculus with water warmed to 20°C to minimise thermal stress from cold water to their core body (Hull 1999). After which, birds were gently inverted, and regurgitated material was collected (Offredo and Ridoux 1986; Quillfeldt 2002; Ridoux and Offredo 1989). Samples were preserved in 90% ethanol and later analysed in the laboratory at the University of Tasmania. To prevent resampling of individuals, a small section of one tail feather was clipped from each bird.

In the laboratory, samples were washed, and prey items were separated individually. Squid and fish abundance were determined from the most significant number of lower or upper squid beaks and left or right otoliths, respectively. Crustacean abundance was quantified as the number of whole bodies or heads with paired eyes. Where possible, prey items were identified to species level. Cephalopod beaks, crustaceans, and sagittal otoliths were identified by comparison to reference atlases (Gomen et al. 2008; Lu and Ickeringill 2002; Poore 2004; Xavier and Cherel 2009) and other species to a reference atlas (Anderson 2001).

#### 2.1.5 Density Surveys

Annual density surveys (chicks per hectare) were conducted on Muttonbird Island during the late chick‐provisioning phase from 2016 to 2018, by counting the number of chicks located within 1 m on either side of randomly placed 50 m transects across the colony (2015–2016; 600 m^2^, 2016–2017; 860 m^2^, 2017–2018; 900 m^2^). Historical data collected before this study were obtained from the NSW National Parks and Wildlife Service (Nesbitt 2014; Nesbitt et al. 2012). For Barunguba, survey data were provided by the authors of a long‐term monitoring programme spanning 1967 to 2018 (Fullagar et al. 2019). At this colony, chick density was estimated by counting chicks within three fixed‐area plots across an area of 1014 m^2^.

### 2.2 Statistical Analysis

All data were processed using the R programming language, version 4.4.0 (R Core Team 2024).

#### 2.2.1 Spatial and Temporal Segregation

Daily positions were estimated using the curve‐fitting method (Lisovski et al. 2019) in the *SGAT* package (Sumner et al. 2009; Wotherspoon et al. 2013). This Bayesian approach applies a Markov Chain Monte Carlo (MCMC) framework to estimate the posterior distribution of locations (Sumner et al. 2009). With locations estimated for each bird (95% CI) from pre‐processed light data using a set of priors, which included a spatial probability mask that excluded locations on land, the average speed of movement between locations was assumed to be Gamma distributed (Sumner 2011).

The location estimates were used to generate gridded density maps (0.1° resolution) with the *raster* package (Hijmans 2020). The location limitations were reduced to a mean of 70.1 ± 23.5 km^2^ based on the area of the 95% quantile using the 95% quantile of primary positions *n* = 39,809, calculated as the mean of the best fit including a fitted generalised additive model line (mean = 70.11 km^2^, SD ±23.54 km^2^, median = 74.46 km^2^, minimum = 16.08 km^2^ and maximum = 170.61 km^2^) (Sumner 2011). Assumptions of normality were checked before statistical comparisons. A Welch *t*‐test was used to assess differences between populations for distances travelled and locations, with significance set at *p* < 0.05 level. Results are reported as mean ± standard deviation.

Maps were created using the *tmap* package (Tennekes 2018) with bathymetry data from the U.S. National Ocean and Atmospheric Administration (NOAA 2021). Movement metrics (distance, direction, and speed) were derived using the *traipse* package (Sumner 2022).

The degree of spatiotemporal overlap in habitat use was obtained by summing the number of locations present in each raster cell. The higher the number of locations and the number of instances a bird was present in a raster cell, the higher the relative value and importance of that raster cell. The area of overlap was derived by calculating the area in square kilometres of each raster cell, using the highest cell value at the 10%, 25%, and 50% quantiles of overlap.

Individual movement was determined by analysing the spatial data, with an individual bird defined as having departed for their winter migration when the first consistent northward movement, exceeding 3° in latitude from their breeding colony. Their return is based on the corresponding southward shift. Pre‐staging detours were identified when individuals moved greater than 4° south in latitude from their breeding colony during the late chick‐provisioning stage, just before their northward migration.

Frontal zone locations were determined from sea surface height (SSH) data using the method of Venables et al. (2012) and the *raadtools* package (Sumner 2025). Sea surface height values were obtained from the Copernicus Marine Environment Monitoring Service (marine.copernicus.eu). Following Chapman et al. (2020) and Venables et al. (2012), the southern extent of the Subtropical Frontal Zone was defined as 0.5 dyn cm with mean dynamic height values matched to the breeding phase dates annually.

#### 2.2.2 Trophic Niche Differentiation

We tested for outliers by plotting residuals against fitted values to assess homogeneity of variance and by plotting residuals against each covariate to evaluate the relationship between predictors and outcomes (Zuur et al. 2010). Two samples were removed from the analysis in the third year for Barunguba. The mean and standard deviation of isotopic values were tested between colonies and years using the *FSA* package version 0.10.0 (Ogle et al. 2025). Differences between colony and years were tested using a Kruskal–Wallis test, followed by Dunn's test for post hoc pairwise comparisons, with *p*‐values adjusted using Bonferroni correction to reduce the risk of Type 1 errors.

The rKIN package (Albeke 2025) was used to analyse δ^13^C and δ^15^N values to calculate Bayesian niche size and overlap. Within rKIN, the kernel utilisation density model, which accommodates extreme outliers, unbalanced samples, and nonparametric data, was chosen. The kernel utilisation density function was used to estimate the isotopic niche width and percentage overlap among years at the 50%, 75%, and 95% contours. The bivariate model sums two separate functions (*x* = δ^13^C and *y* = δ^15^N) across observed data points, with the extent determined by the minimum and maximum values of δ^13^C and δ^15^N, calculated on a grid larger than that of the observational samples (Eckrich et al. 2020).

The utilisation distributions of δ^13^C and δ^15^N were estimated using minimum contour sizes from a non‐parametric estimator (Balzani et al. 2020; Eckrich et al. 2020; Hickerson et al. 2019). Niche size was quantified based on the extent of spacing within the bivariate isotopic space and dietary position overlap between the two colonies (Eckrich et al. 2020). Larger niche space estimates were interpreted as indicative of a broader variety of prey and habitats used (Lois et al. 2020). The position of the subtropical front was based on Jaeger, Lecomte, et al. (2010).

Frequency of occurrence of prey items was calculated as the number of stomachs in which each prey item was present, expressed as a percentage of the total number of stomachs examined (Lima‐Junior and Goitein 2001). Prey abundance, diversity, and evenness were quantified using the Shannon diversity index (Magurran 2004). To avoid bias, the number of birds of each species was divided by the total number of samples in which that prey item occurred (Petta et al. 2020; Shannon and Weaver 1949).

The equitable evenness index (*H*/*H*_max_; Magurran 2004) was used to compare diet samples. Values approaching zero indicate a predator that consumes only one or a few prey items of the total available. In contrast, values approaching one indicate consumption of a broad variety of prey items.

#### 2.2.3 Population Dynamics and Breeding Success

The commencement of the breeding phases was identified by analysing the spatial data from individual birds. Chick density survey data were used to compare per‐hectare variances between populations. They were analysed with the *ggpmisc* package by fitting a linear regression line with a 95% confidence interval and calculating Pearson's correlation coefficient.

## 3 Results

### 3.1 Spatial and Temporal Segregation

**FIGURE 2:**
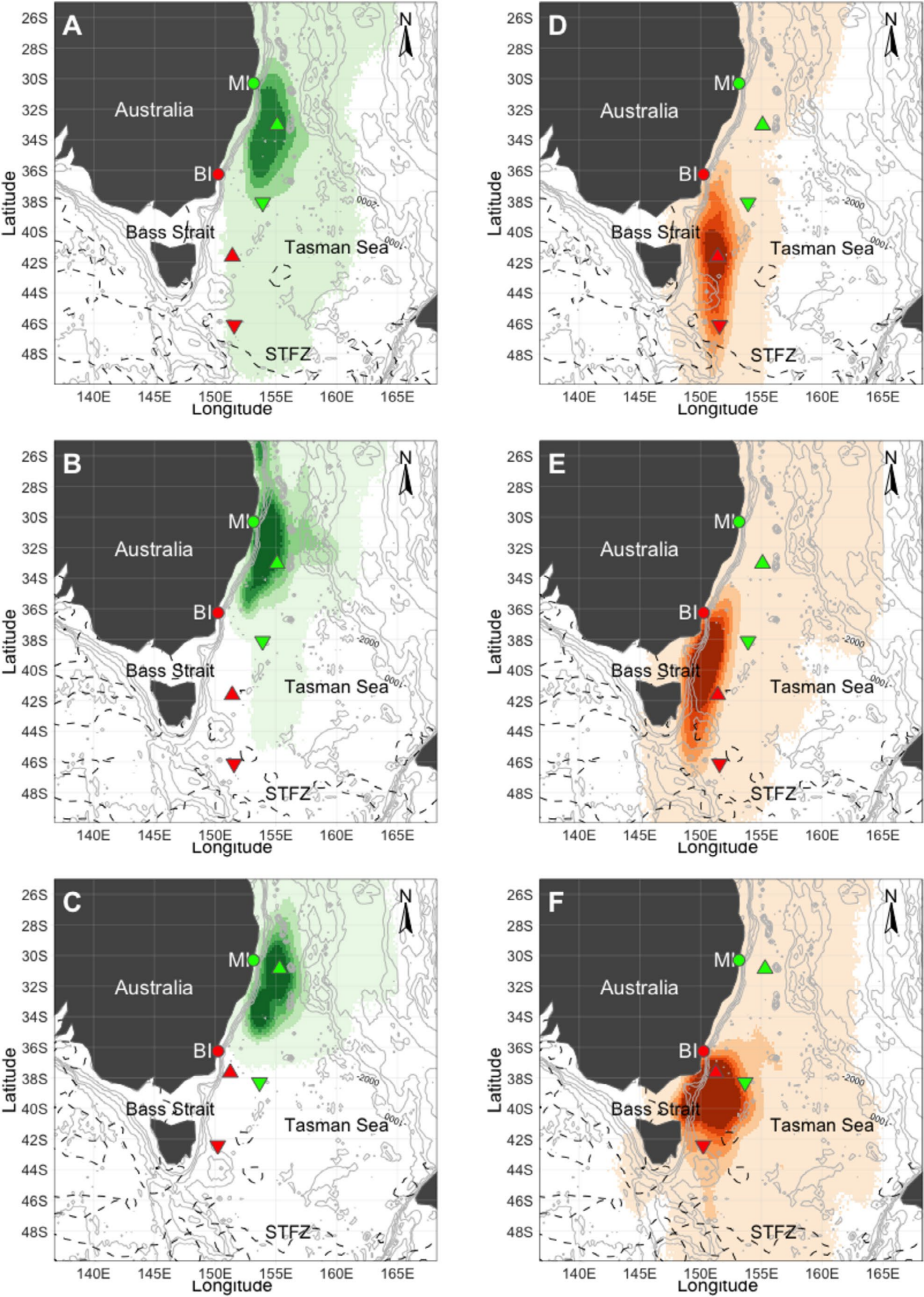
At‐sea distribution and spatiotemporal segregation during chick‐provisioning from Muttonbird Island (MI) and Barunguba (BI). (A–C) MI 2016–2018 (*n* = 12, 5, 4); (D–F), BI 2016–2018 (*n* = 7, 17, 11). Green (MI) and red (BI) circles represent the mapped population; green (MI) and red (BI) north‐pointing triangles represent the mean maximum location north; and green (MI) and red (BI) south‐pointing triangles represent the absolute mean maximum southern location. The dashed black line represents the approximate location of the subtropical frontal zone.

During the breeding phase, individuals from both colonies foraged extensively across the Tasman Sea, exhibiting strong spatial segregation throughout all study years (Figures 1 and 2). Individuals from Muttonbird Island foraged over larger core areas (2015–2016; 392,358 km^2^, 2016–2017; 528,098 km^2^, 2017–2018; 417,185 km^2^) than those from Barunguba (2015–2016; 123,873 km^2^, 2016–2017; 215,025 km^2^, 2017–2018; 270,690 km^2^) at the 10% decile in all years (Table 1), although this pattern was not observed at the 25% and 50% deciles during 2017–2018. At the 10% decile, there was no overlap in the first 2 years, and only 4% (28,457 km^2^) overlap occurred in 2017–2018. At the 25% and 50% deciles, overlap never exceeded 22% across all years (128,412−1,150,632 km^2^ depending on decile) (Table 1).

**TABLE 1:** Summary of spatiotemporal analysis as a decile or quantile of the total area wedge‐tailed shearwaters were present in each raster cell during the incubation and chick‐rearing phase for Muttonbird (MI) and Barunguba (BI) islands. The area and percentage of overlap are based on the cells in which individuals were most frequently present.

Year and decile %	% Overlap	Overlap area km^2^	Core area used km^2^	
			MI	BI
2015–2016	*n* = 20			
10%	0%	0	392,358	123,873
25%	8%	128,412	973,125	613,967
50%	22%	722,637	1,948,434	1,283,499
2016–2017	*n* = 20			
10%	0%	0	528,098	215,025
25%	7%	203,866	1,908,353	1,108,704
50%	11%	1,150,632	7,927,371	2,277,163
2017–2018	*n* = 25			
10%	4%	28,457	417,185	270,690
25%	22%	538,408	1,036,230	1,400,832
50%	19%	956,269	2,117,529	2,871,170

During the incubation phase, habitat use was clearly segregated, with Muttonbird Island individuals mainly using waters off Australia's east coast (Figure 1). In contrast, Barunguba individuals travelled further south into Bass Strait. The mean distance from the colony was greater for the temperate Barunguba population during the incubation phase in 2017–2018 (420 km ±118 vs. 297 km ±187, *p*‐value < 0.001), but not in the second year (305 km ±131 vs. 317 km ±152, *p*‐value 0.56; Table 2), with both colonies undertaking shorter trips during the incubation phase for all years.

**TABLE 2:** Summary of mean ± SD and Welch *t*‐test between population and year, for distances travelled of wedge‐tailed shearwaters during the incubation and chick‐provisioning stages, for Muttonbird (MI) and Barunguba (BI) islands, the number of individuals denoted in brackets ().

	Chick‐provisioning		Incubation		Incubation and chick‐provisioning
	Distance travelled from colony km (mean ± SD)	*p*	Distance travelled from colony km (mean ± SD)	*p*	Southern maximum distance travelled km (mean ± SD)
MI					
2015–2016	439 ± 237	< 0.001	354 ± 146	—	1036 ± 384 (14)
2016–2017	400 ± 227	< 0.001	317 ± 152	0.56	1079 ± 168 (6)
2017–2018	329 ± 152	< 0.001	297 ± 187	< 0.001	1050 ± 428 (6)
BI					
2015–2016	608 ± 304	< 0.001	—	—	1033 ± 260 (6)
2016–2017	516 ± 299	< 0.001	305 ± 131	0.56	1186 ± 242 (17)
2017–2018	480 ± 314	< 0.001	420 ± 118	< 0.001	936 ± 439 (23)

During the chick‐provisioning phase, habitat use was more widespread than during incubation (Figures 1 and 2), with a high degree of spatial segregation between colonies. Muttonbird Island individuals concentrated their foraging in waters southeast and slightly northeast of the colony (Figure 2A–C), while Barunguba individuals ranged extensively south, exploiting the Bass Strait eastern edge as well as waters east and southeast of Tasmania (Figure 2D–F).

Combined tracking data during the incubation and chick provisioning phase showed that the mean location of Barunguba individuals was significantly more southerly (BI; −38.62° S, 151.14° E, *p*‐value < 0.001) compared to Muttonbird Island individuals (MI; 31.91° S, 155.12° E) (Table 3). The southernmost mean location for all years was further south for Barunguba individuals (−44.07° S 150.31° E), which was further south than that of Muttonbird Island individuals (−38.39° S 153.83° E), with some Barunguba individuals travelling as far as the subtropical frontal zone (Figure 2D–F). A Welch *t*‐test showed that the mean foraging locations differed significantly between populations across all years, *p*‐value < 0.001 (Table 3).

**TABLE 3:** Summary of mean ± SD and Welch *t*‐test between population and year, for the southern maximum and pre‐staging detour locations of wedge‐tailed shearwaters during the incubation and chick‐provisioning stages, for Muttonbird (MI) and Barunguba (BI) islands, the number of individuals denoted in brackets ().

Incubation and chick‐provisioning				
	Mean ± SD location	*p*‐value	Southern maximum location (mean ± SD)	Pre‐staging detour location south (mean ± SD)
MI				
2015–2016	−33.04° S 155.09° E (12)	< 0.001	−38.11° S 153.90° E (12)	−38.93° S ±4.01 (14)
2016–2017	−30.65° S 154.27° E (5)	< 0.001	−37.41° S 153.67° E (5)	−40.85° S (1)
2017–2018	−30.87° S 155.30° E (6)	< 0.001	−38.30° S 153.65° E (6)	—
All years	−31.91° S 155.12° E (23)		−38.39° S 153.83° E (23)	−39.89° S ±4.01 (15)
BI				
2015–2016	−41.63° S 151.40° E (7)	< 0.001	−46.12° S 151.55° E (7)	−46.12° S ±2.85 (7)
2016–2017	−38.83° S 150.85° E (17)	< 0.001	−45.40° S 149.93° E (17)	−45.71° S ±1.86 (17)
2017–2018	−38.62° S 151.14° E (23)	< 0.001	−42.41° S 150.21° E (23)	−44.61° S ±2.44 (17)
All years	−38.62° S 151.14° E (47)		−44.07° S 150.31° E (47)	−45.48° S ±2.38 (34)

**FIGURE A1:**
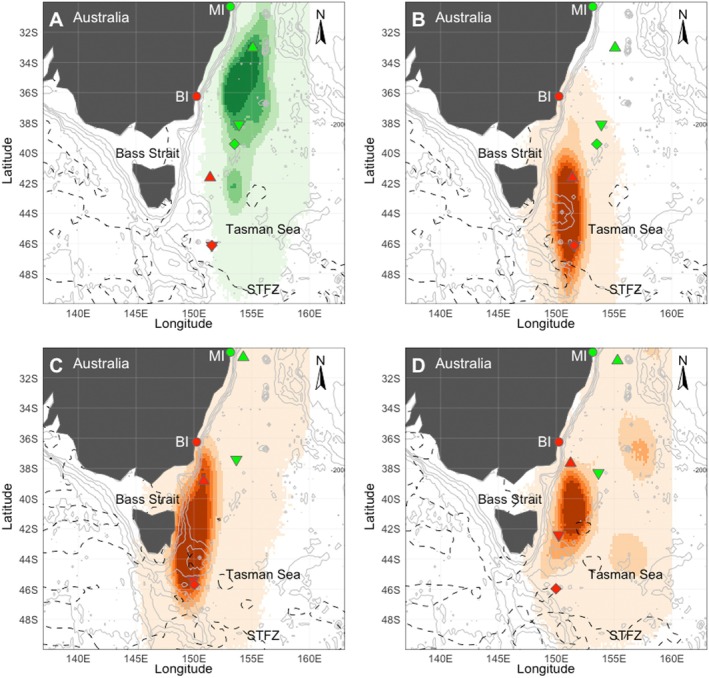
At‐sea distribution and spatiotemporal segregation of wedge‐tailed shearwaters during a pre‐staging detour from Muttonbird Island (MI) and Barunguba (BI). (A) MI 2015–2016 (*n* = 10); Panels: (B–D) BI 2015–2016 (*n* = 7), 2016–2017 (*n* = 16) and 2017–2018 (*n* = 9). Green (MI) and red (BI) circles denote colony locations. North‐pointing triangles indicate the mean northern maximum location; south‐pointing triangles indicate the absolute mean southern maximum location. The dashed black line represents the approximate position of the subtropical frontal zone.

Directly after breeding but before migrating to the Northern Hemisphere, most tracked Barunguba individuals (34 of 37) undertook a pre‐staging detour to the edge of the subtropical frontal zone in waters southeast of Tasmania (Figure A1B–D). Ten of the 12 tracked individuals from Muttonbird Island undertook a similar southward pre‐staging detour in 2015–2016, but only one of five did so in 2016–2017, and none did so in 2017–2018 (Figure A1A). Because Barunguba individuals consistently undertook this southward pre‐staging trip before commencing their winter migration to the Philippine Sea, their mean northward departure date was 23 ± 14 days later than that of Muttonbird Island birds (Table 4).

**TABLE 4:** Annual life cycle of wedge‐tailed shearwaters by population. Values are the mean start and finish dates ± variation in days from the average as a minimum or maximum. Data were obtained from tracking data, and the 2015–2016 period was not representative of a full year because devices failed between 3 and 6 months after deployment. Data were collected from Muttonbird (2015–2018) and Barunguba (2016–2019) islands.

	*n*	Arrival date at breeding location	Incubation start date	Chick‐provisioning start date	Departure date from breeding location
MI					
2015–2016	11		24 Nov ±8	13 Jan ±10	17 Mar ±13
2016–2017	5	28 Aug ±17	24 Nov ±2	11 Jan ±2	5 Apr ±13
2017–2018	3	29 Aug ±0	21 Nov ±0	10 Jan ±5	12 Mar ±2
All years		28 Aug ±10	24 Nov ±4	12 Jan ±3	16 Mar ±14
BI					
2015–2016	6				15 Apr ±7
2016–2017	15	6 Sep ±8		14 Jan ±2	11 Apr ±13
2017–2018	11	2 Sep ±12	22 Nov ±6	15 Jan ±5	9 Apr ±22
2018–2019	13	7 Sep ±13	21 Nov ±4	14 Jan ±7	9 Apr ±15
All years		5 Sep ±6	22 Nov ±3	14 Jan ±3	8 Apr ±14

### 3.2 Trophic Niche Differentiation

**FIGURE 3:**
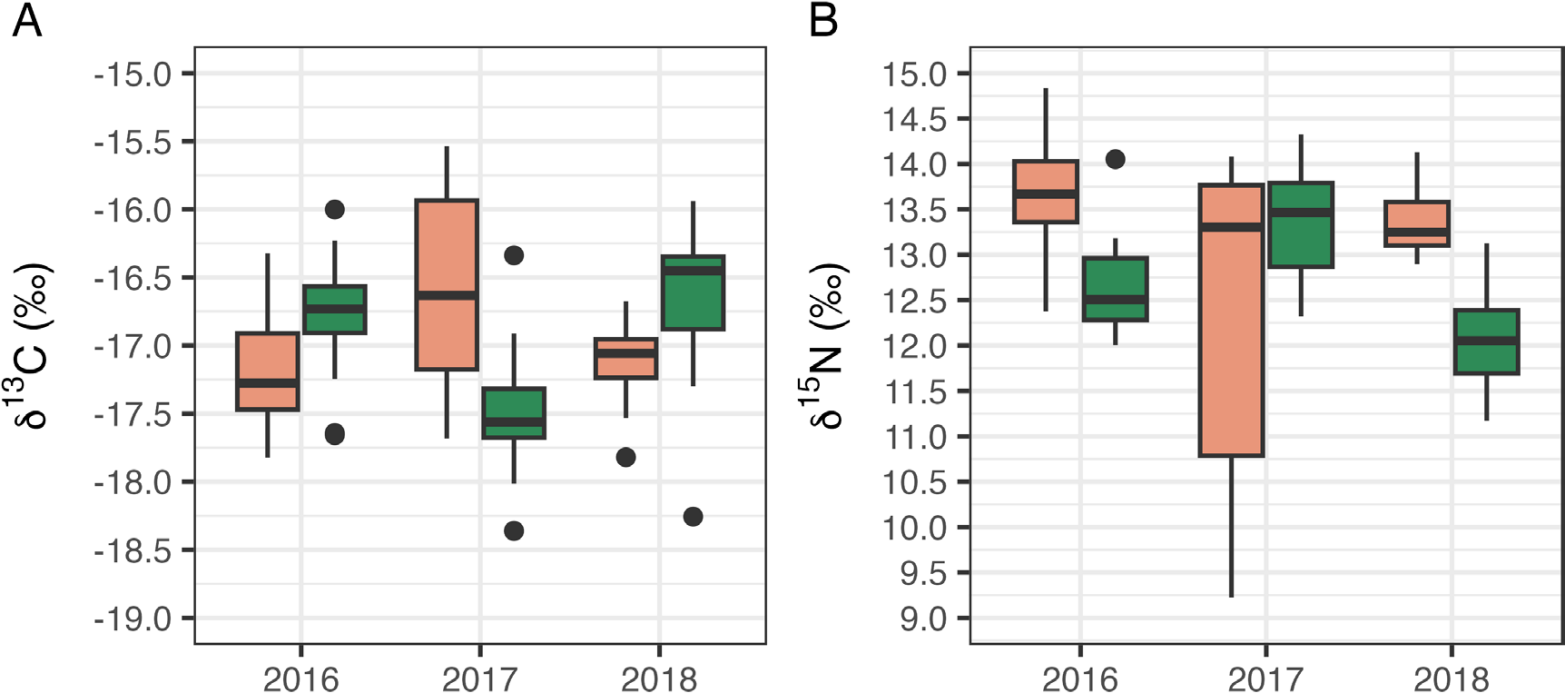
Stable isotope values (A: δ13C and B: δ15N) of wedge‐tailed shearwater chick feathers collected during chick‐provisioning (*n* = 191). Green is Muttonbird Island (MI); orange is Barunguba (BI).

Mean δ^13^C values differed among years during the chick‐provisioning stage, with the Muttonbird Island population exhibiting higher values than Barunguba in 2016 and 2018 but lower values in 2017 (Table 5, Figure 3). For δ^15^N values, individuals from Muttonbird Island had lower mean values than Barunguba in 2016 and 2018, but higher values in 2017. The highest mean δ^15^N values occurred in 2016 for the Barunguba population (Table 5). Overall, inter‐population differences were significant for both isotopes in all years except δ^15^N in 2017 (Kruskal–Wallis, *χ* ^2^ = 1.77, *p* = 0.18). Within populatios, isotope values for Muttonbird Island varied significantly among years for both δ^13^C (*χ* ^2^ = 44.58, *p* < 0.001) and δ^15^N (*χ* ^2^ = 42.97, *p* < 0.001), with all year‐pair comparisons significant (*p* < 0.001). For Barunguba, isotope values also differed significantly among years for δ^13^C (*χ* ^2^ = 16.80, *p* < 0.001) and δ^15^N (*χ* ^2^ = 12.89, *p* < 0.001), although pairwise differences were primarily driven by the 2016 values (*p* < 0.001) (Table 5).

**TABLE 5:** Inter‐annual comparisons of mean ± SD, minimum (min) and maximum (max) values of carbon (δ^13^C) and nitrogen (δ^15^N) stable isotope values from feathers obtained from wedge‐tailed shearwater chicks during the chick‐provisioning stage from Muttonbird (MI) and Barunguba (BI) islands. Values correspond to *p*‐value adjusted using the Bonferroni‐adjusted value.

	*n*	δ^13^C				δ^15^N			
		Mean ± SD	Min	Max	*p*	Mean ± SD	Min	Max	*p*
MI									
2016	35	−16.77 ± 0.35	−16.00	−17.66	< 0.001	12.61 ± 0.45	12.00	14.05	< 0.001
2017	30	−17.52 ± 0.40	−16.34	−18.36	< 0.001	13.32 ± 0.57	12.32	14.33	< 0.001
2018	29	−16.62 ± 0.48	−15.94	−18.26	< 0.001	12.10 ± 0.54	11.17	13.13	< 0.001
BI									
2016	42	−17.19 ± 0.35	−16.32	−17.82	< 0.001	13.69 ± 0.56	12.38	14.84	< 0.001
2017	30	−16.57 ± 0.68	−15.54	−17.68	0.36	12.29 ± 1.76	9.23	14.08	0.02
2018	26	−17.08 ± 0.25	−16.68	−17.82	0.08	13.34 ± 0.33	12.90	14.13	1.00

**FIGURE 4:**
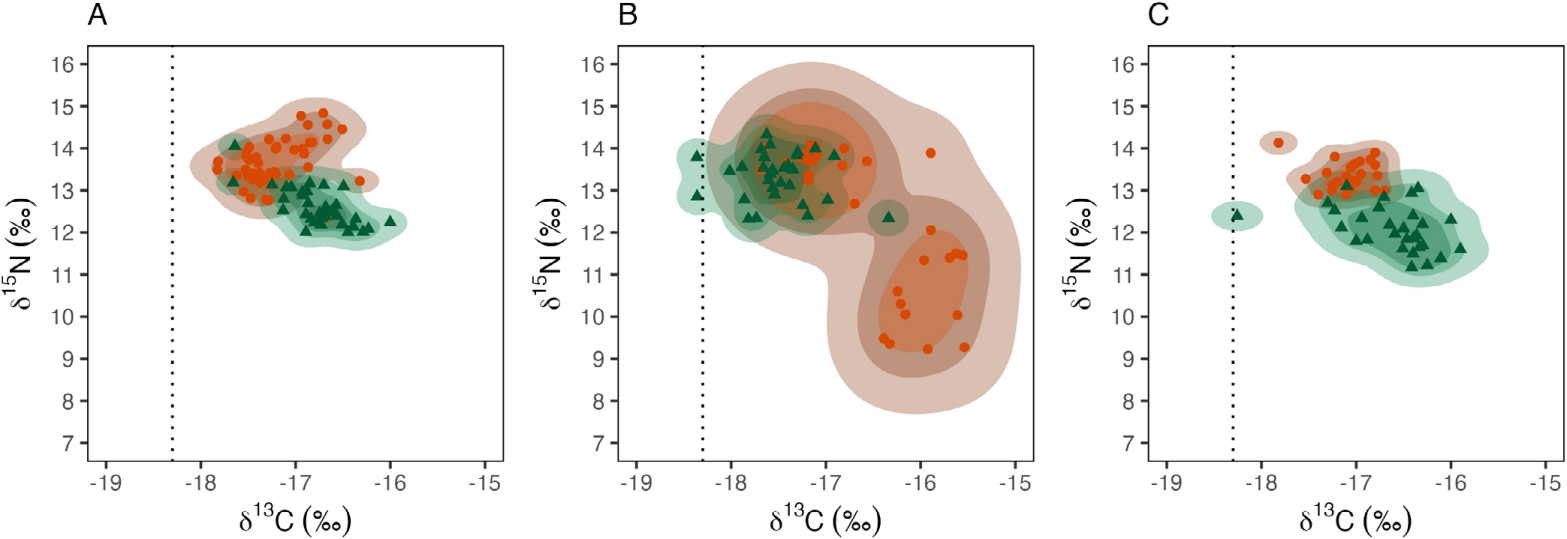
Isotopic niche area estimates generated using kernel utilisation density (KUD) for wedge‐tailed shearwater chicks from Muttonbird Island (MI, green triangles) and Barunguba (BI, orange circles). (A) (2016, *n* = 77); (B) (2017, *n* = 60); and (C) (2018, *n* = 54). The dashed black line represents the approximate location of the subtropical frontal zone.

The kernel utilisation density model of δ^13^C and δ^15^N values from chick feathers revealed clear separation in isotopic niche space between populations in 2016 and 2018 (Figure 4). In contrast, niches overlapped in 2017, when Barunguba exhibited the largest niche area across all years and populations and overlapped with Muttonbird Island (Figure 4). Muttonbird Island had the smallest niche area in both 2016 (MI; 0.59–2.67, BI; 0.88–3.77) and 2017 (MI; 0.85–3.71, BI; 4.91–18.19), whereas Barunguba had the smallest niche area in 2018 (MI; 1.05–3.78, BI; 0.36–1.52) (Table 6).

**TABLE 6:** Isotopic niche space estimates generated using kernel utilisation density (KUD) model for wedge‐tailed shearwaters from Muttonbird (MI) and Barunguba (BI) islands for carbon (δ^13^C) and nitrogen (δ^15^N) obtained from feathers, at 50%, 75% and 95% KUD contour levels.

	2016			2017			2018		
	50%	75%	95%	50%	75%	95%	50%	75%	95%
MI	0.59	1.19	2.67	0.85	1.83	3.71	1.05	2.06	3.78
BI	0.88	1.74	3.77	4.91	9.63	18.19	0.36	0.71	1.52

The core isotopic niche area estimated using kernel utilisation density did not overlap between Muttonbird Island and Barunguba at the 50% contour in 2016. Only minimal overlap occurred at broader contours, 0.10% at the 75% and 0.44% at the 95% contours (Table A1; Figure 4A). A similar pattern occurred in 2018, with no overlap at the 50% contour and limited overlap at the 75% (0.07%) and 95% (0.18%) contours (Table A1, Figure 4C). In contrast, overlap was greatest in 2017, occurring across all contour levels (0.87%, 0.85%, and 0.86% at the 50%, 75%, and 95% contours, respectively) (Table A1, Figure 4B).

Across years, Muttonbird Island generally had a smaller isotopic niche area and lower interannual variability (0.59–3.78) than Barunguba (0.36–18.19) (Figure 4). Overall, the kernel utilisation density model indicated that Muttonbird Island had the smallest isotopic niche area in 2016 and 2017, whereas Barunguba had the smalleset niche area in 2018 (Figure 4, Table 6).

A total of 245 individual prey items were identified from the stomach contents of 57 adults (29 January–18 March in 2016). Prey included cephalopods, fish, crustacea, and a single species of hydrozoa, *Velella velella*. Although not all taxa could be identified to the species level, two species dominated the samples: the striped flying squid ( *Eucleoteuthis luminosa* ) and a leatherjacket ( *Paramonacanthus filicauda* ).

**FIGURE 5:**
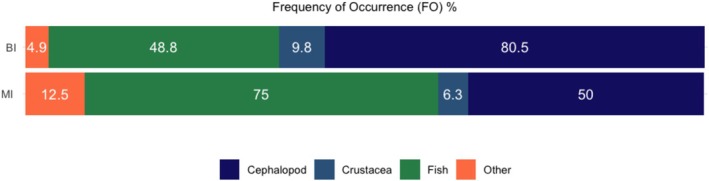
Frequency of occurrence (FO%) of major prey groups recovered from adult wedge‐tailed shearwaters during the chick‐provisioning (2016). Muttonbird Island (MI, *n* = 16) and Barunguba (BI, *n* = 41). Values represent the proportion of stomach samples containing each prey item.

Prey composition differed markedly between colonies. Samples from Muttonbird Island had a high frequency of occurrence of fish (75.0%), dominated by a leatherjacket (*P. filicauda*). In contrast, those from Barunguba had a high frequency of occurrence of cephalopods (80.5%), dominated by the striped flying squid ( *E. luminosa* ) (Figure 5).

Dietary diversity indices supported this difference. The Shannon diversity index and evenness were both higher for Muttonbird Island (*H′* = 1.231; *E* = 0.633) than for Barunguba (*H′* = 0.867; *E* = 0.395). These results indicate that Muttonbird Island birds consumed a more diverse and evenly distributed range of prey. Meanwhile, individuals from Barunguba exhibited a greater reliance on a smaller number of prey types.

### 3.3 Population Dynamics and Breeding Success

Adults arrived at the breeding colonies approximately one week apart from 28 August (±10 days) on Muttonbird Island and from 5 September (±6 days) on Barunguba (Table 4). The incubation phase was synchronous across populations, beginning 21–24 November (±4 days) in all years. The onset of chick‐provisioning was synchronous across both colonies, occurring on 10–15 January (±3 days). Northern migration timing differed by 23 ± 14 days between populations, reflecting a pre‐staging movement undertaken by individuals from Barunguba.

**FIGURE 6:**
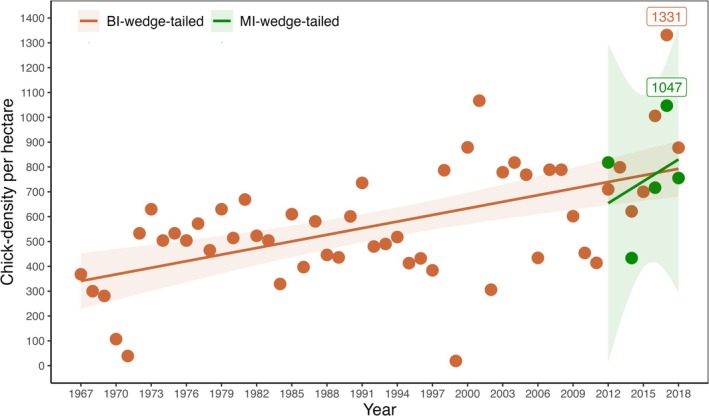
Temporal trends in wedge‐tailed shearwater chick density (chicks per hectare). Linear regression with 95% confidence intervals and Pearson's correlation coefficient shown. Barunguba; 1967 to 2018; Muttonbird Island; 2012–2018 (excluding 2013 and 2015). Labels indicate the highest densities recorded.

Wedge‐tailed shearwater chick density varied among years at both colonies, with the highest densities recorded in the 2016–2017 breeding season for both populations (MI; 1047 ± 180 chicks per hectare; BI; 1331 ± 257 chicks per hectare; Figure 6). Over the past 52 years, chick density on Barunguba has increased significantly (*r* = 0.55, *p* < 0.001, *n* = 52). Although Muttonbird Island exhibited a similar upward trend in chick density (*r* = 0.32, *p* = 0.60, *n* = 5), there are insufficient data points to draw firm conclusions. Across years, the subtropical population at Muttonbird Island had a lower mean density (0.075 chicks m^‐^^2^, equivalent to 750 chicks ha^‐^^1^), than the temperate population at Barunguba (0.093 chicks m^‐^^2^, 930 chicks ha^‐^^1^).

## 4 Discussion

This study provides a novel comparison of wedge‐tailed shearwaters from two distinct climatic zones, including a temperate population at the southern extent of this species' range. We combined tracking data, stable isotope ratios, diet composition, and chick density surveys to reveal clear segregation in spatial use and isotopic niches between two disjunct populations during the breeding phase.

The temperate Barunguba population consistently exploited areas further south than previously documented (~5.5° S), reaching the subtropical frontal zone and likely targeting more predictable prey resources. In contrast, the subtropical Muttonbird Island population ranged further north and east across a broader area, suggesting that prey resources were patchier and less predictable.

Some phenological differences were evident, with Muttonbird Island adults arriving ~7 days earlier and departing ~23 days sooner than Barunguba adults. With a pre‐staging detour documented in individuals from Barunguba, who consistently travelled south to a mean location of −45.48° S ±2.38 before commencing their winter migration to the Philippine Sea, compared with Muttonbird Island individuals, −39.89° S ±4.01.

Bulk isotopic analysis indicated minimal overlap between populations in 2016 and 2018, but not in 2017, when Barunguba exhibited a broader and more variable niche breadth. Dietary analysis revealed that Muttonbird Island individuals consumed a higher proportion of fish, predominantly leatherjackets, with a generalist diet. In contrast, Barunguba individuals preferred squid, particularly striped flying squid, and had a more specialised diet.

Chick density surveys showed similar interannual trends across colonies, but consistently lower overall densities at Muttonbird Island than at Barunguba.

### 4.1 Spatial and Temporal Segregation

The results revealed a high degree of habitat partitioning, minimal spatiotemporal overlap, and variation in distances travelled during the chick‐provisioning phase across all years, although this pattern was not observed during the incubation phase in 2016–2017. Some variation in winter arrival and departure dates were confirmed, despite similar timing of incubation and chick‐provisioning phases. The sub‐tropical colony on Muttonbird Island consistently used a larger foraging area across most years, except in 2017–2018 at > 25% decile. This broader foraging range may reflect less predictable or stable prey resources compared with populations in more temperate regions. As with other shearwater species, prey availability and timing are likely to drive differences in foraging behaviour (Granadeiro et al. 1998; Miller 2018; Weimerskirch et al. 2020). Studies from Lord Howe Island and New Caledonia demonstrate wedge‐tailed shearwaters switching from unimodal to bimodal foraging strategies in response to declining prey availability, highlighting their behavioural flexibility (Miller 2018; Weimerskirch et al. 2020). While this study did not directly investigate whether Muttonbird Island birds adopted this type of foraging behaviour, the spatiotemporal patterns suggest birds may have exhibited some degree of foraging flexibility during the 2016–2017 study year.

Consistent with findings from tropical regions (McDuie et al. 2015; Weimerskirch et al. 2020), Muttonbird Island birds typically travelled 329–439 km from the breeding site. Individuals from the temperate Barunguba colony generally travelled slightly further, not dissimilar to observations of wedge‐tailed shearwaters on Lord Howe Island (Miller 2018).

Tracking data showed across all years, Barunguba adults foraged at more southerly latitudes than Muttonbird Island adults. Because carbon values are typically more depleted at southerly latitudes, individuals foraging farther south will exhibit lower δ^13^C values (Jaeger, Connan, et al. 2010). Such differences were evident in the first and third years, but not the second, when Muttonbird Island individuals potentially foraged further inshore, where prey is relatively more enriched in carbon (Bond and Jones 2009). This pattern aligns with similar studies of divergent foraging strategies, which is advantageous for this species (Miller 2018; Peck et al. 2008; Peck and Congdon 2005).

During the chick‐provisioning phase, Barunguba adults consistently travelled towards the subtropical frontal zone before departing for their wintering grounds in the Philippine Sea. This frontal zone provides access to higher concentrations and reliable prey resources such as myctophid fish, and is a key foraging habitat for many marine predators (Bost et al. 2009; Foo et al. 2019). Frontal zones are areas consistently targeted by seabirds due to the higher abundance of macroplankton biomass, resulting from higher primary production, attracting a larger number of prey and predators (Bost et al. 2009). This enables seabirds to gain weight more quickly, as prey is more predictable and abundant at frontal zones, in preparation for the energetically costly migration to their winter habitat in the Philippine Sea (Warnock 2010). A comparable pre‐staging strategy has been documented in short‐tailed shearwaters, which travel south to 60°–70° S and remain for 26.1 ± 16.4 days before migrating to the North Pacific Ocean (Carey et al. 2014). Stopover behaviour during migration is a typical strategy used by seabirds to replenish fat reserves before long‐distance movements, including in species such as the sooty shearwater (Hedd et al. 2012), Manx shearwater ( *Puffinus puffinus* ) (Guilford et al. 2009), little auk ( *Alle alle* ) (Mosbech et al. 2012), and Sabine's gull ( *Larus sabini* ) (Stenhouse et al. 2012).

To date, no other study has documented a southerly pre‐staging strategy in wedge‐tailed shearwaters prior to their northward migration. On Heron Island, individuals were observed using stopover sites while migrating to their core nonbreeding habitat in Micronesia (McDuie 2016). However, several other studies have not reported this behaviour in wedge‐tailed shearwaters (Miller et al. 2018; Surman et al. 2018; Weimerskirch et al. 2020).

### 4.2 Trophic Niche Differentiation

Stable isotope analysis of chick feather samples revealed prey selectivity in both temperate and subtropical populations, except for Barunguba individuals in 2017. During that year, the temperate population showed evidence of exploiting a broader range of prey types and habitats, suggesting that adults provisioned their chicks with a more diverse prey type. This pattern may reflect high variability in the timing, type, or location of available prey. Similar flexibility has been documented in wedge‐tailed shearwaters from Lord Howe and Heron islands (McDuie et al. 2015; Miller 2018; Weimerskirch et al. 2020), where such flexibility supports chick growth and fledging success (Hodum and Hobson 2000). These interannual shifts highlight a degree of foraging adaptability in the temperate Barunguba population. Notably, in 2017, Barunguba chicks occupied two distinct and relatively large isotopic niche areas (Figure 4B), consistent with provisioning from a broader range of prey types, potentially sourced from different habitats.

For both Muttonbird Island and Barunguba, the highest chick densities recorded occurred in 2017, coinciding with the broadest isotopic niche breadth observed for the temperate population observed in this study. Greater prey availability in that year may have enabled higher‐than‐average breeding participation and improved fledgling success. The long‐term increase in the Barunguba colony density may also be linked to its proximity to productive habitats associated with cooler waters and oceanographic fronts. The subtropical frontal zone may therefore not only be fuelling the increasing density but also contribute to the recent expansion of the species' breeding range to Gabo Island, ~150 km south of Barunguba (De Almeida E Silva 2025; Hunter 2020).

Diet sampling further suggested the Barunguba population could be exploiting more highly concentrated and predictable prey resources to provision their chick. Seabirds commonly use olfactory cues to locate prey (Bost et al. 2009), and there is evidence that some predators also detect temperature gradients to locate frontal zones where prey are more abundant (Chapman et al. 2020). The diets observed in this study were dominated by fish and cephalopods, consistent with previous studies of wedge‐tailed shearwaters (Gagne et al. 2018; Komura et al. 2018; Ravache et al. 2020; Spear et al. 2007). As expected, both prey types played a key role in both populations. Whilst limitations exist in using direct prey samples to infer diet over short timescales, and the small sample size obtained in this study, the results nonetheless provide valuable insight into the demography and prey sources of wedge‐tailed shearwaters in southeastern Australia (Buckland et al. 2017; Pethybridge et al. 2018; Polito et al. 2011). Importantly, this study presents dietary results from a temperate population.

### 4.3 Population Dynamics and Breeding Success

Wedge‐tailed shearwaters are colonial breeders that often nest alongside sympatric species with overlapping breeding phases (Ravache et al. 2020). On Barunguba, they breed with both short‐tailed shearwaters and, in smaller numbers, sooty shearwaters ( *A. grisea* ). Overlap in breeding phases among these species may increase competition for quality burrow sites and prey resources (Ravache et al. 2020).

Temporal segregation partly mitigates this overlap with wedge‐tailed shearwaters arriving earlier (mid‐August to early September) than short‐tailed (October) and sooty shearwaters (late September to early October) (Jones et al. 2003). Earlier access to burrows likely provides wedge‐tailed shearwaters with a competitive advantage, potentially reducing the breeding success of later‐arriving species (Hunter 2020; Quintana and Yorio 1998; Ramos et al. 1997). Similar competitive interactions have been observed between wedge‐tailed shearwaters and the similarly sized Tahiti petrel ( *Pseudobulweria rostrata* ) in New Caledonia (Villard et al. 2006). Long‐term monitoring on Barunguba has shown an increase in wedge‐tailed shearwater chick density per hectare alongside a decline in short‐tailed shearwaters, indicating an interspecific shift in species composition (Fullagar et al. 2019). Warmer climatic conditions during breeding may be favouring wedge‐tailed shearwaters, as seen in Western Australia, where the Rottnest Island population doubled over a 10‐year period (Bancroft et al. 2004). Recent attempts to breed further south, on Gabo Island (De Almeida E Silva 2025; Hunter 2020), suggest that conditions in temperate regions may be favouring wedge‐tailed shearwaters to expand their breeding range south, potentially at the detriment of sympatric species.

During this study, the average chick density was lower on Muttonbird Island (0.075 m^2^) than on Barunguba (0.093 m^2^), likely due to multiple factors, including poorer burrow quality, altered food webs, and more frequent extreme weather events associated with climate change (Swanson et al. 2023). On Barunguba, catastrophic rainfall events in 1970 and 1999 caused widespread burrow collapse and breeding failures in wedge‐tailed and short‐tailed shearwaters (Tiller et al. 2013). Burrows on Muttonbird Island tend to be shallower, with limited vegetation, making them particularly vulnerable to weather events and prone to collapse. The burrowing activities of introduced rats ( *Rattus rattus* and *R. norvegicus* ) and house mice ( *Mus musculus* ) could also be destabilising shearwater burrows further (Muttonbird Island Nature Reserve Plan of Management 2009), although this study did not evaluate this impact. Introduced species, particularly black rats, have been shown to have ecologically devastating impacts on smaller seabirds (< 300 g body weight), especially burrow‐nesting species (Banks and Hughes 2012; Jones et al. 2008). They prey on a broad range of sources, including seabird eggs, chicks, and adults, and are highly adaptable to fluctuations in prey availability (Jones et al. 2008). Although the present study did not assess the impacts of introduced species on Muttonbird Island, a study on Broughton Island found a decline in shearwater density was not likely due to the removal of invasive species but rather at‐sea impacts on prey resources (Carlile et al. 2022). However, as a consequence of poorer burrow quality, chicks are more exposed to predators such as the Australian Raven ( *Corvus coronoides* ) and white‐bellied sea eagle ( *Haliaeetus leucogaster* ), as well as to weather extremes that increase thermoregulation demands (Swanson et al. 2023).

On Heron Island during the breeding season of 2001–2002, reproductive failure of wedge‐tailed shearwaters (Smithers et al. 2003) was linked to poor nutrient availability, a common feature of oligotrophic tropical marine ecosystems (Smithers et al. 2003). The lower chick densities on Muttonbird Island compared to those on Barunguba may actually be reflecting the lower productivity of the waters where Muttonbird Island birds forage. Adults from Muttonbird Island may not only return with less food for their chicks but also expend more energy doing so due to consistently having to forage across larger areas.

### 4.4 Climate Change Implications and Conservation Relevance

Both populations demonstrated high foraging and spatiotemporal flexibility, indicating that wedge‐tailed shearwaters have the capability and adaptability to shift into new, more productive habitats beyond their current southern range. This flexibility is critical in the face of climate change, which is expected to alter oceanographic conditions and the dynamics of frontal zones (Gervais et al. 2021). Southeastern Australia is particularly vulnerable, predicted to warm at three to four times the global average (Gervais et al. 2021; Phillips et al. 2020). Such changes may facilitate range expansion of tropical and subtropical species, enabling wedge‐tailed shearwaters to extend their breeding distribution further south.

Understanding species habitat and spatiotemporal use, and trophic niches, is critical for predicting how they may adapt to environmental change. The temperate waters of southeastern Australia are recognised marine biodiversity hotspots (Wernberg et al. 2011), yet are experiencing population‐level shifts in demographics, species interactions, and life cycles under the influence of climate change and an intensifying East Australian Current (Carroll et al. 2016; Doney et al. 2012; Jenouvrier et al. 2017).

Our findings show that spatial segregation and divergent trophic niches between temperate and subtropical populations of wedge‐tailed shearwaters help minimise intraspecific competition during the breeding phase. By exploiting waters further south than previously recorded, these populations are accessing highly productive areas in marine frontal zones, highlighting this species' strong foraging adaptability. Such flexibility is likely to be a key factor enabling wedge‐tailed shearwaters to adapt to future climate change and contribute to higher breeding success into the future for populations located in temperate Australia.

This adaptability suggests that wedge‐tailed shearwaters have the capacity to expand into new habitats and establish colonies beyond their current southern range as oceanographic conditions shift. However, such range expansions could exacerbate competition with sympatric seabird species, potentially reshaping community composition in temperate breeding colonies in southeastern Australia.

## Author Contributions

**Penny E. Beaver:** conceptualization (equal), data curation (lead), formal analysis (lead), funding acquisition (equal), investigation (lead), methodology (lead), project administration (lead), resources (equal), validation (lead), visualization (equal), writing – original draft (lead), writing – review and editing (equal). **Nicholas Carlile:** conceptualization (equal), data curation (equal), formal analysis (equal), funding acquisition (equal), investigation (equal), methodology (equal), resources (equal), supervision (equal), visualization (equal), writing – review and editing (equal). **Michael D. Sumner:** formal analysis (equal), software (equal). **Mary‐Anne Lea:** conceptualization (equal), funding acquisition (equal), methodology (equal), project administration (equal), resources (equal), supervision (lead), visualization (equal), writing – original draft (equal), writing – review and editing (equal).

## Funding

The NSW Office of Environment and Heritage and Birdlife Australia provided funding.

## Ethics Statement

All animal handling and instrumentation were conducted under the following research permits: NSW Office of Environment and Heritage AEC021028/02; and University of Tasmania Animal Ethics Committee permits A15502 and A17679.

## Conflicts of Interest

The authors declare no conflicts of interest.

## Data Availability

Data available from the Birdlife Seabird Tracking Database: https://data.seabirdtracking.org/dataset/2313 and https://data.seabirdtracking.org/dataset/2312. R Code from: https://github.com/pebeaver/Wedge‐tailed_shearwater_habitat_use.

## Appendix A

**TABLE A1:** Percent overlap estimates of isotopic niche space using kernel utilisation density (KUD) method, for wedge‐tailed shearwaters from Muttonbird (MI) and Barunguba (BI) islands for carbon δ^13^C and nitrogen δ^15^N obtained from feathers, at 50%, 75% and 95% contour levels.

% overlap		BI			MI		
		50%	75%	95%	50%	75%	95%
2016	**BI**						
	50%	—	—	—	0.00	0.04	0.41
	75%	0.51	—	—	0.01	0.07	0.37
	95%	0.23	0.46	—	0.08	0.14	0.37
	**MI**						
	50%	0.00	0.01	0.53	—	—	—
	75%	0.03	0.10	0.44	0.50	—	—
	95%	0.13	0.24	0.52	0.22	0.44	—
2017	**BI**						
	50%	—	—	—	0.17	0.29	0.42
	75%	0.51	—	—	0.10	0.19	0.31
	95%	0.27	0.53	—	0.05	0.12	0.21
	MI						
	50%	0.87	—	—	—	—	—
	75%	0.67	0.85	—	0.45	—	—
	95%	0.46	0.67	0.86	0.21	0.47	—
2018	**BI**						
	50%	—	—	—	0.00	0.10	0.71
	75%	0.45	—	—	0.00	0.17	0.60
	95%	0.19	0.42	—	0.00	0.16	0.39
	**MI**						
	50%	0.00	0.00	0.01	—	—	—
	75%	0.02	0.07	0.15	0.52	—	—
	95%	0.06	0.12	0.18	0.26	0.50	—

*Note:* A dash (—) indicates 100% overlap.

## References

[ece373158-bib-0001] Albeke, S. E. 2025. “rKIN (Kernel) Isotope Niche Estimation. R Package Version 1.0.4.” https://CRAN.R‐project.org/package=rKIN.

[ece373158-bib-0002] Anderson, D. T. 2001. Invertebrate Zoology. 2nd ed. Oxford University Press.

[ece373158-bib-0003] Balzani, P. , R. E. Gozlan , and P. J. Haubrock . 2020. “Overlapping Niches Between Two Co‐Occurring Invasive Fish: The Topmouth Gudgeon *Pseudorasbora parva* and the Common Bleak *Alburnus alburnus* .” Journal of Fish Biology 97, no. 5: 1385–1392. 10.1111/jfb.14499. 33460088

[ece373158-bib-0004] Bancroft, W. J. , M. J. Garkaklis , and J. D. Roberts . 2004. “Continued Expansion of the Wedge‐Tailed Shearwater, *Puffinus pacificus*, Nesting Colonies on Rottnest Island, Western Australia.” Emu—Austral Ornithology 104, no. 1: 79–82. 10.1071/MU03010.

[ece373158-bib-0005] Banks, P. B. , and N. K. Hughes . 2012. “A Review of the Evidence for Potential Impacts of Black Rats ( *Rattus rattus* ) on Wildlife and Humans in Australia.” Wildlife Research 39, no. 1: 78–88. 10.1071/WR11086.

[ece373158-bib-0006] Barrett, R. T. , C. J. Camphuysen , T. Anker‐Nilssen , et al. 2007. “Diet Studies of Seabirds: A Review and Recommendations.” ICES Journal of Marine Science 64, no. 9: 1675–1691.

[ece373158-bib-0007] Berlincourt, M. , and J. P. Y. Arnould . 2015. “Breeding Short‐Tailed Shearwaters Buffer Local Environmental Variability in South‐Eastern Australia by Foraging in Antarctic Waters.” Movement Ecology 3, no. 1: 1–16. 10.1186/s40462-015-0044-7. 26236479 PMC4522076

[ece373158-bib-0008] Bond, A. L. , and I. Jones . 2009. “A Practical Introduction to Stable‐Isotope Analysis for Seabird Biologists: Approaches, Cautions and Caveats.” Marine Ornithology 37, no. 3: 183–188.

[ece373158-bib-0009] Bontempo, L. , F. Ceppa , L. Ziller , et al. 2014. “Comparison of Methods for Stable Isotope Ratio (δ^13^C, δ^15^N, δ^2^H, δ^18^O) Measurements of Feathers.” Methods in Ecology and Evolution 5: 363–371.

[ece373158-bib-0010] Bost, C. A. , C. Cotté , F. Bailleul , et al. 2009. “The Importance of Oceanographic Fronts to Marine Birds and Mammals of the Southern Oceans.” Journal of Marine Systems 78, no. 3: 363–376. 10.1016/j.jmarsys.2008.11.022.

[ece373158-bib-0011] Buckland, A. , R. Baker , N. Loneragan , and M. Sheaves . 2017. “Standardising Fish Stomach Content Analysis: The Importance of Prey Condition.” Fisheries Research 196: 126–140. 10.1016/j.fishres.2017.08.003.

[ece373158-bib-0012] Carey, M. J. , R. A. Phillips , J. R. D. Silk , and S. A. Shaffer . 2014. “Trans‐Equatorial Migration of Short‐Tailed Shearwaters Revealed by Geolocators.” Emu—Austral Ornithology 114, no. 4: 352–359. 10.1071/MU13115.

[ece373158-bib-0313] Carlile, N. , S. Callaghan , and M. Garrard . 2022. “Expansion of Ardenna Shearwater Breeding Colonies on Broughton Island After Eradication of the European Rabbit and Black Rat.” Corella 46: 27–30.

[ece373158-bib-0013] Carlile, N. , A. Harris , and C. Lloyd . 2020. “Seabird Islands No. 2/1: Montague Island, New South Wales—Additional Breeding Seabirds.” Corella 44: 71–73.

[ece373158-bib-0014] Carroll, G. , R. Harcourt , I. Jonsen , J. D. Everett , and D. Slip . 2016. “High Sea Surface Temperatures Driven by a Strengthening Current Reduce Foraging Success by Penguins.” Scientific Reports 6: 22236. 10.1038/srep22236. 26923901 PMC4770590

[ece373158-bib-0015] Catry, T. , J. A. Ramos , M. Le Corre , and R. A. Phillips . 2009. “Movements, At‐Sea Distribution and Behaviour of a Tropical Pelagic Seabird: The Wedge‐Tailed Shearwater in the Western Indian Ocean.” Marine Ecology Progress Series 391: 231–292.

[ece373158-bib-0016] Cetina‐Heredia, P. , M. Roughan , E. van Sebille , and M. A. Coleman . 2014. “Long‐Term Trends in the East Australian Current Separation Latitude and Eddy Driven Transport.” Journal of Geophysical Research: Oceans 119, no. 7: 4351–4366. 10.1002/2014jc010071.

[ece373158-bib-0017] Chapman, C. C. , M.‐A. Lea , A. Meyer , J.‐B. Sallée , and M. A. Hindell . 2020. “Defining Southern Ocean Fronts and Their Influence on Biological and Physical Processes in a Changing Climate.” Nature Climate Change 10, no. 3: 209–219. 10.1038/s41558-020-0705-4.

[ece373158-bib-0018] Cherel, Y. , K. A. Hobson , and H. Weimerskirch . 2000. “Using Stable‐Isotope Analysis of Feathers to Distinguish Moulting and Breeding Origins of Seabirds.” Oecologia 122, no. 2: 155–162. 10.2307/4222528. 28308369

[ece373158-bib-0019] Corman, A.‐M. , B. Mendel , C. C. Voigt , and S. Garthe . 2016. “Varying Foraging Patterns in Response to Competition? A Multicolony Approach in a Generalist Seabird.” Ecology and Evolution 6, no. 4: 974–986. 10.1002/ece3.1884. 26941940 PMC4761771

[ece373158-bib-0020] Cory, F. , A. Wilson , D. Priddel , N. Carlile , and N. Klomp . 2011. “Eradication of the House Mouse (*Mus musculus*) From Montague Island, New South Wales, Australia.” Ecological Management & Restoration 12, no. 2: 102–109. 10.1111/j.1442-8903.2011.00583.x.

[ece373158-bib-0021] De Almeida E Silva, N. 2025. “Influences of Environmental Change on Short‐Tailed Shearwater Population Through Multi‐Level Monitoring.” Doctor of Philosophy, Deakin University, Australia.

[ece373158-bib-0022] Doney, S. C. , M. Ruckelshaus , J. E. Duffy , et al. 2012. “Climate Change Impacts on Marine Ecosystems.” Annual Review of Marine Science 4: 11–37. 10.1146/annurev-marine-041911-111611. 22457967

[ece373158-bib-0023] Eckrich, C. A. , S. E. Albeke , E. A. Flaherty , R. T. Bowyer , and M. Ben‐David . 2020. “rKIN: Kernel‐Based Method for Estimating Isotopic Niche Size and Overlap.” Journal of Animal Ecology 89, no. 3: 757–771. 10.1111/1365-2656.13159. 31799690

[ece373158-bib-0024] Floyd, R. B. , and N. M. Swanson . 1982. “Wedge‐Tailed Shearwaters on Muttonbird Island: An Estimate of the Breeding Success and the Breeding Population.” Emu—Austral Ornithology 82, no. 5: 244–250.

[ece373158-bib-0025] Foo, D. , C. McMahon , M. Hindell , S. Goldsworthy , and F. Bailleul . 2019. “Influence of Shelf Oceanographic Variability on Alternate Foraging Strategies in Long‐Nosed Fur Seals.” Marine Ecology Progress Series 615: 189–204.

[ece373158-bib-0026] Fullagar, P. J. , C. Davey , D. Priddel , and M. Crowley . 2019. 60th Annual Assessment of Shearwater Breeding Success on Montagu Island, 19–27 March 2019.

[ece373158-bib-0027] Gagne, T. O. , K. D. Hyrenbach , M. E. Hagemann , and K. S. Van Houtan . 2018. “Trophic Signatures of Seabirds Suggest Shifts in Oceanic Ecosystems.” Science Advances 4, no. 2: eaao3946. 10.1126/sciadv.aao3946. 29457134 PMC5812733

[ece373158-bib-0028] Gervais, C. R. , C. Champion , and G. T. Pecl . 2021. “Species on the Move Around the Australian Coastline: A Continental‐Scale Review of Climate‐Driven Species Redistribution in Marine Systems.” Global Change Biology 27, no. 14: 3200–3217. 10.1111/gcb.15634. 33835618 PMC8251616

[ece373158-bib-0029] Gomen, M. , D. Bray , and R. Kuiter , eds. 2008. Fishes of Australia's Southern Coast. Reed New Holland, Museum Victoria.

[ece373158-bib-0030] Granadeiro, J. P. , M. Nunes , M. C. Silva , and R. W. Furness . 1998. “Flexible Foraging Strategy of Cory's Shearwater, *Calonectris diomedea* , During the Chick‐Rearing Period.” Animal Behaviour 56: 1169–1176. 9819333 10.1006/anbe.1998.0827

[ece373158-bib-0031] Guilford, T. , J. Meade , J. Willis , et al. 2009. “Migration and Stopover in a Small Pelagic Seabird, the Manx Shearwater *Puffinus puffinus* : Insights From Machine Learning.” Proceedings of the Biological Sciences 276, no. 1660: 1215–1223. 19141421 10.1098/rspb.2008.1577 PMC2660961

[ece373158-bib-0032] Hedd, A. , W. A. Montevecchi , H. Otley , R. A. Phillips , and D. A. Fifield . 2012. “Trans‐Equatorial Migration and Habitat Use by Sooty Shearwaters *Puffinus griseus* From the South Atlantic During the Nonbreeding Season.” Marine Ecology Progress Series 449: 277–290.

[ece373158-bib-0033] Hickerson, B. T. , B. M. Maitland , and A. W. Walters . 2019. “Effects of Multiple Nonnative Fish on an Imperiled Cyprinid, Hornyhead Chub.” Transactions of the American Fisheries Society 148, no. 6: 1132–1145. 10.1002/tafs.10203.

[ece373158-bib-0034] Hijmans, R. J. 2020. “raster: Geographic Data Analysis and Modeling (Version R Package Version 3.4‐5).” https://CRAN.R‐project.org/package=raster.

[ece373158-bib-0035] Hobson, K. A. 1999. “Tracing Origins and Migration of Wildlife Using Stable Isotopes: A Review.” Oecologia 120: 314–326. 28308009 10.1007/s004420050865

[ece373158-bib-0036] Hobson, K. A. 2007. “Isotopic Tracking of Migrant Wildlife.” In Stable Isotopes in Ecology and Environmental Science, edited by R. Michener and K. Lajtha , 2nd ed., 155–175. Blackwell Publishing.

[ece373158-bib-0037] Hodum, P. J. , and K. A. Hobson . 2000. “Trophic Relationships Among Antarctic Fulmarine Petrels: Insights Into Dietary Overlap and Chick Provisioning Strategies Inferred From Stable‐Isotope (δ^15^N and δ^13^C) Analyses.” Marine Ecology Progress Series 198: 273–281. 10.3354/meps198273.

[ece373158-bib-0038] Hull, C. L. 1999. “Comparison of the Diets of Breeding Royal ( *Eudyptes schlegeli* ) and Rockhopper ( *Eudyptes chrysocome* ) Penguins on Macquarie Island Over Three Years.” Journal of Zoology 247, no. 4: 507–529. 10.1111/j.1469-7998.1999.tb01013.x.

[ece373158-bib-0039] Hunter, C. 2020. “Niche Segregation in Sympatric Short‐Tailed and Wedge‐Tailed Shearwaters.” Honours Thesis, Deakin University.

[ece373158-bib-0040] Jaeger, A. , M. Connan , P. Richard , and Y. Cherel . 2010. “Use of Stable Isotopes to Quantify Seasonal Changes of Trophic Niche and Levels of Population and Individual Specialisation in Seabirds.” Marine Ecology Progress Series 401: 269–277. 10.3354/meps08380.

[ece373158-bib-0041] Jaeger, A. , V. J. Lecomte , H. Weimerskirch , P. Richard , and Y. Cherel . 2010. “Seabird Satellite Tracking Validates the Use of Latitudinal Isoscapes to Depict Predators' Foraging Areas in the Southern Ocean.” Rapid Communications in Mass Spectrometry 24, no. 23: 3456–3460. 10.1002/rcm.4792. 21072802

[ece373158-bib-0042] Jenouvrier, S. , M. Desprez , R. Fay , et al. 2017. “Climate Change and Functional Traits Affect Population Dynamics of a Long‐Lived Seabird.” Journal of Animal Ecology 87: 906–920. 10.1111/1365-2656.12827 29931768

[ece373158-bib-0043] Jones, C. , S. Bettany , H. Moller , D. Fletcher , P. Lyver , and J. de Cruz . 2003. “Burrow Occupancy and Productivity at Coastal Sooty Shearwater (*Puffinus griseus*) Breeding Colonies, South Island, New Zealand: Can Mark–Recapture Be Used to Estimate Burrowscope Accuracy?” Wildlife Research 30: 377–388.

[ece373158-bib-0044] Jones, H. P. , B. R. Tershy , E. S. Zavaleta , et al. 2008. “Severity of the Effects of Invasive Rats on Seabirds: A Global Review.” Conservation Biology 22, no. 1: 16–26. 10.1111/j.1523-1739.2007.00859.x. 18254849

[ece373158-bib-0045] Komura, T. , H. Ando , K. Horikoshi , H. Suzuki , and Y. Isagi . 2018. “DNA Barcoding Reveals Seasonal Shifts in Diet and Consumption of Deep‐Sea Fishes in Wedge‐Tailed Shearwaters.” PLoS One 13, no. 4: e0195385. 29630670 10.1371/journal.pone.0195385 PMC5891018

[ece373158-bib-0046] Lima‐Junior, S. E. , and R. Goitein . 2001. “A New Method for the Analysis of Fish Stomach Contents.” Acta Scientiarum 23, no. 2: 421–424.

[ece373158-bib-0047] Lisovski, S. , S. Bauer , M. Briedis , et al. 2019. “Light‐Level Geolocator Analyses: A User's Guide.” Journal of Animal Ecology 89, no. 1: 221–236. 10.1111/1365-2656.13036. 31190329

[ece373158-bib-0048] Lois, N. A. , L. Campagna , U. Balza , et al. 2020. “Metapopulation Dynamics and Foraging Plasticity in a Highly Vagile Seabird, the Southern Rockhopper Penguin.” Ecology and Evolution 10, no. 7: 3346–3355. 10.1002/ece3.6127. 32273992 PMC7141044

[ece373158-bib-0049] Lu, C. C. , and R. Ickeringill . 2002. Cephalopod Beak Identification and Biomass Estimation Techniques: Tools for Diettary Studies of Southern Australian Finfishes (Vol. Reports (6) Museum Victoria. Fisheries Research and Development Corporation). Museum Victoria.

[ece373158-bib-0050] Magurran, A. E. 2004. Measuring Biological Diversity. Blackwell Publishing.

[ece373158-bib-0051] Marchant, S. , and P. J. Higgins . 1990. Handbook of Australian and New Zealand and Antarctic Birds (Vol. 1: Ratites to Ducks, Part B Australian Pelican to Ducks). Oxford University Press.

[ece373158-bib-0052] McCormack, S. A. , R. Trebilco , J. Melbourne‐Thomas , J. L. Blanchard , E. A. Fulton , and A. Constable . 2019. Using Stable Isotope Data to Advance Marine Food Web Modelling. Kluwer Academic Publishing.

[ece373158-bib-0053] McDuie, F. 2016. “Critical Foraging Locations and Oceanographic Relationships for Great Barrier Reef Breeding Seabirds.” Doctor of Philosphy, James Cook University.

[ece373158-bib-0054] McDuie, F. , and B. C. Congdon . 2016. “Trans‐Equatorial Migration and Non‐Breeding Habitat of Tropical Shearwaters: Implications for Modelling Pelagic Important Bird Areas.” Marine Ecology Progress Series 550: 219–234. 10.3354/meps11713.

[ece373158-bib-0055] McDuie, F. , S. J. Weeks , A. K. Miller , and B. C. Congdon . 2015. “Breeding Tropical Shearwaters Use Distant Foraging Sites When Self Provisioning.” Marine Ornithology 43: 123–129.

[ece373158-bib-0056] Miller, M. G. R. 2018. “Foraging Niche Specialisation and Resource Use in Tropical Seabirds: Implications for Management.” Doctor of Philosophy, James Cook University.

[ece373158-bib-0057] Miller, M. G. R. , N. Carlile , J. Scutt Phillips , F. McDuie , and B. C. Congdon . 2018. “Importance of Tropical Tuna for Seabird Foraging Over a Marine Productivity Gradient.” Marine Ecology Progress Series 586: 233–249. 10.3354/meps12376.

[ece373158-bib-0058] Mosbech, A. , K. L. Johansen , N. I. Bech , et al. 2012. “Inter‐Breeding Movements of Little Auks *Alle alle* Reveal a Key Post‐Breeding Staging Area in the Greenland Sea.” Polar Biology 35, no. 2: 305–311. 10.1007/s00300-011-1064-4.

[ece373158-bib-0107] Muttonbird Island Nature Reserve Plan of Management . 2009. NSW National Parks and Wildlife Service. Department of the Environment and Climate Change NSW.

[ece373158-bib-0059] Nesbitt, B. 2014. Muttonbird Island & North Solitary Island Nature Reserves, Wedge‐Tailed Shearwater (Mutton Bird) Burrow Survey Results 2013/14 Breeding Season. Retrieved from North Coast Region.

[ece373158-bib-0060] Nesbitt, B. , S. Townley , D. Brown , and J. Smith . 2012. Muttonbird Island & North Solitary Island Nature Reserves, Wedge‐Tailed Shearwater (Mutton Bird) Burrow Survey 2011/12 Breeding Season. Retrieved from North Coast Region.

[ece373158-bib-0061] Newsome, S. D. , C. M. d. Rio , S. Bearhop , and D. L. Phillips . 2007. “A Niche for Isotopic Ecology.” Frontiers in Ecology and the Environment 5, no. 8: 429–436.

[ece373158-bib-0062] NOAA . 2021. “National Geophysical Data Centre, GEODAS Grid Translator Design a Grid.” https://www.ngdc.noaa.gov/mgg/gdas/gd_designagrid.html.

[ece373158-bib-0063] Offredo, C. , and V. Ridoux . 1986. “The Diet of Emperor Penguins *Aptenodytes forsteri* in Adelie Land, Antarctica.” Ibis 128, no. 3: 409–413. 10.1111/j.1474-919X.1986.tb02690.x.

[ece373158-bib-0064] Ogle, D. H. , J. C. Doll , A. P. Wheeler , and A. Dinno . 2025. “FSA: Simple Fisheries Stock Assessment Methods (Version 0.10.0).” https://CRAN.R‐project.org/package=FSA.

[ece373158-bib-0065] Peck, D. R. , W. J. Bancroft , and B. C. Congdon . 2008. “Morphological and Molecular Variation Within an Ocean Basin in Wedge‐Tailed Shearwaters ( *Puffinus pacificus* ).” Marine Biology 153, no. 6: 1113–1125. 10.1007/s00227-007-0883-x.

[ece373158-bib-0066] Peck, D. R. , and B. C. Congdon . 2005. “Colony‐Specific Foraging Behaviour and Co‐Ordinated Divergence of Chick Development in the Wedge‐Tailed Shearwater *Puffinus pacificus* .” Marine Ecology Progress Series 299: 289–296.

[ece373158-bib-0067] Peck, D. R. , B. V. Simthers , A. K. Krockenberger , and B. C. Congdon . 2004. “Sea Surface Temperature Constrains Wedge‐Tailed Shearwater Foraging Success Within Breeding Seasons.” Marine Ecology Progress Series 281: 259–266.

[ece373158-bib-0068] Pethybridge, H. R. , C. A. Choy , J. J. Polovina , and E. A. Fulton . 2018. “Improving Marine Ecosystem Models With Biochemical Tracers.” Annual Review of Marine Science 10: 199–228. 10.1146/annurev-marine-121916-063256 29298140

[ece373158-bib-0069] Petta, J. C. , O. N. Shipley , S. P. Wintner , G. Cliff , M. L. Dicken , and N. E. Hussey . 2020. “Are You Really What You Eat? Stomach Content Analysis and Stable Isotope Ratios Do Not Uniformly Estimate Dietary Niche Characteristics in Three Marine Predators.” Oecologia 192: 1111–1126. 10.1007/s00442-020-04628-6. 32179976

[ece373158-bib-0070] Phillips, L. R. , G. Carroll , I. Jonsen , R. Harcourt , and M. Roughan . 2020. “A Water Mass Classification Approach to Tracking Variability in the East Australian Current.” Frontiers in Marine Science 7: 365. 10.3389/fmars.2020.00365.

[ece373158-bib-0071] Polito, M. J. , W. Z. Trivelpiece , N. J. Karnovsky , E. Ng , W. P. Patterson , and S. D. Emslie . 2011. “Integrating Stomach Content and Stable Isotope Analyses to Quantify the Diets of Pygoscelid Penguins.” PLoS One 6, no. 10: e26642. 10.1371/journal.pone.0026642. 22053199 PMC3203888

[ece373158-bib-0072] Polito, M. J. , W. Z. Trivelpiece , W. P. Patterson , N. J. Karnovsky , C. S. Reiss , and S. D. Emslie . 2015. “Contrasting Specialist and Generalist Patterns Facilitate Foraging Niche Partitioning in Sympatric Populations of Pygoscelis Penguins.” Marine Ecology Progress Series 519: 221–237. 10.3354/meps11095.

[ece373158-bib-0073] Poore, G. C. B. 2004. Marine Decapod Crustacea of Southern Australia: A Guide to Identification. Museum Victoria.

[ece373158-bib-0074] Quillfeldt, P. 2002. “Seasonal and Annual Variation in the Diet of Breeding and Non‐Breeding Wilson's Storm‐Petrels on King George Island, South Shetland Islands.” Polar Biology 25, no. 3: 216–221. 10.1007/s00300-001-0332-0.

[ece373158-bib-0075] Quillfeldt, P. , R. A. R. McGill , and R. W. Furness . 2005. “Diet and Foraging Areas of Southern Ocean Seabirds and Their Prey Inferred From Stable Isotopes: Review and Case Study of Wilson's Storm‐Petrel.” Marine Ecology Progress Series 295: 295–304.

[ece373158-bib-0076] Quintana, F. , and P. Yorio . 1998. “Competition for Nest Sites Between Kelp Gulls ( *Larus dominicanus* ) and Terns (*Sterna maxima* and *S. eurygnatha* ) in Patagonia.” Auk 115, no. 4: 1068–1071.

[ece373158-bib-0077] R Core Team . 2024. R: A Language and Environment for Statistical Computing. R Foundation for Statistical Computing, Vienna, Austria. https://www.R‐project.org/.

[ece373158-bib-0078] Ramos, J. A. , L. R. Monteiro , E. Sola , and Z. Moniz . 1997. “Characteristics and Competition for Nest Cavities in Burrowing Procellariiformes.” Condor: Ornithological Applications 99, no. 3: 634–641. 10.2307/1370475.

[ece373158-bib-0079] Ravache, A. , K. Bourgeois , H. Weimerskirch , et al. 2020. “Behavioral and Trophic Segregations Help the Tahiti Petrel to Cope With the Abundance of Wedge‐Tailed Shearwater When Foraging in Oligotrophic Tropical Waters.” Scientific Reports 10, no. 1: 15129. 10.1038/s41598-020-72206-0. 32934324 PMC7492251

[ece373158-bib-0080] Ridgeway, K. , and K. Hill . 2009. “The East Australian Current.” In A Marine Climate Change Impacts and Adaptions Report Card for Australia 2009. NCCARF Publications.

[ece373158-bib-0081] Ridgway, K. R. , and S. D. Ling . 2023. “Three Decades of Variability and Warming of Nearshore Waters Around Tasmania.” Progress in Oceanography 215: 103046. 10.1016/j.pocean.2023.103046.

[ece373158-bib-0082] Ridoux, V. , and C. Offredo . 1989. “The Diets of Five Summer Breeding Seabirds in Adélie Land, Antarctica.” Polar Biology 9, no. 3: 137–145. 10.1007/bf00297168.

[ece373158-bib-0083] Schultz, M. A. , and N. I. Klomp . 2000. “Chick‐Provisioning Behaviour of Two Shearwaters Breeding in South‐Eastern Australia.” Austral Ecology 25: 219–326.

[ece373158-bib-0084] Shannon, C. E. , and W. Weaver . 1949. The Mathematical Theory of Communication. University of Illinois Press.

[ece373158-bib-0085] Smithers, B. V. , D. R. Peck , A. K. Krockenberger , and B. C. Congdon . 2003. “Elevated Sea‐Surface Temperature, Reduced Provisioning and Reproductive Failure of Wedge‐Tailed Shearwaters ( *Puffinus pacificus* ) in the Southern Great Barrier Reef, Australia.” Marine and Freshwater Research 54: 973–977.

[ece373158-bib-0086] Spear, L. B. , D. G. Ainley , and W. A. Walker . 2007. Foraging Dynamics of Seabirds in the Eastern Tropical Pacific Ocean. Vol. 35. Cooper Ornithological Society.

[ece373158-bib-0087] Stenhouse, I. J. , C. Egevang , and R. A. Phillips . 2012. “Trans‐Equatorial Migration, Staging Sites and Wintering Area of Sabine's Gulls *Larus sabini* in the Atlantic Ocean.” Ibis 154, no. 1: 42–51. 10.1111/j.1474-919X.2011.01180.x.

[ece373158-bib-0088] Sumner, M. D. 2011. “The Tag Location Problem.” Doctor of Philosophy, University of Tasmania, Institute for Marine and Antarctic Studies.

[ece373158-bib-0089] Sumner, M. D. 2022. “traipse: Shared Tools for Tracking Data (Version R Package Version 0.3.0).” https://CRAN.R‐project.org/package=traipse.

[ece373158-bib-0090] Sumner, M. D. 2025. “raadfiles: File Database Management for ‘raadtools’ (Version R Package Version 0.1.4.9013).” https://github.com/australianantarcticdivision/raadfiles.

[ece373158-bib-0091] Sumner, M. D. , S. J. Wotherspoon , and M. A. Hindell . 2009. “Bayesian Estimation of Animal Movement From Archival and Satellite Tags.” PLoS One 4, no. 10: e7324. 10.1371/journal.pone.0007324. 19823684 PMC2758548

[ece373158-bib-0092] Surman, C. A. , L. W. Nicholson , and R. A. Phillips . 2018. “Distribution and Patterns of Migration of a Tropical Seabird Community in the Eastern Indian Ocean.” Journal of Ornithology 159, no. 3: 867–877. 10.1007/s10336-018-1556-x.

[ece373158-bib-0093] Suthers, I. M. , J. W. Young , M. E. Baird , et al. 2011. “The Strengthening East Australian Current, Its Eddies and Biological Effects—An Introduciton and Overview.” Deep Sea Research, Part II: Topical Studies in Oceanography 58, no. 5: 538–546.

[ece373158-bib-0094] Swanson, N. , N. Vaughan , N. Belling , and L. Roman . 2023. “Post‐Fledging Survival of Wedge‐Tailed Shearwaters Is Linked to Pre‐Fledge Mass, Which Has Decreased Over 40 Years.” Marine Ecology 44, no. 6: e12776. 10.1111/maec.12776.

[ece373158-bib-0095] Tennekes, M. 2018. “Tmap: Thematic Maps in R.” Journal of Statistical Software 84, no. 6: 1–39. 10.18637/jss.v084.i06.30450020

[ece373158-bib-0096] Thompson, D. R. , L. G. Torres , G. A. Taylor , et al. 2015. “Stable Isotope Values Delineate the Non‐Breeding Distributions of Sooty Shearwaters *Puffinus griseus* in the North Pacific Ocean.” Marine Ecology Progress Series 521: 277–282.

[ece373158-bib-0097] Tiller, C. J. , N. I. Klomp , P. J. Fullagar , and P. C. Heyligers . 2013. “Catastrophic Breeding Failure Caused by Heavy Rainfall in a Shearwater Colony.” Marine Ornithology 41: 97–99.

[ece373158-bib-0098] Venables, H. , M. P. Meredith , A. Atkinson , and P. Ward . 2012. “Fronts and Habitat Zones in the Scotia Sea.” Deep‐Sea Research Part II 59: 14–24. 10.1016/j.dsr2.2011.08.012.

[ece373158-bib-0099] Villard, P. , S. Dano , and V. Bretagnolle . 2006. “Morphometrics and the Breeding Biology of the Tahiti Petrel *Pseudobulweria rostrata* .” Ibis 148, no. 2: 285–291. 10.1111/j.1474-919X.2006.00528.x.

[ece373158-bib-0100] Warnock, N. 2010. “Stopping vs. Staging: The Difference Between a Hop and a Jump.” Journal of Avian Biology 41, no. 6: 621–626. 10.1111/j.1600-048X.2010.05155.x.

[ece373158-bib-0101] Weimerskirch, H. , S. de Grissac , A. Ravache , et al. 2020. “At‐Sea Movements of Wedge‐Tailed Shearwaters During and Outside the Breeding Season From Four Colonies in New Caledonia.” Marine Ecology Progress Series 633: 225–238. 10.3354/meps13171.

[ece373158-bib-0102] Wernberg, T. , B. D. Russell , P. J. Moore , et al. 2011. “Impacts of Climate Change in a Global Hotspot for Temperate Marine Biodiversity and Ocean Warming.” Journal of Experimental Marine Biology and Ecology 400, no. 1: 7–16. 10.1016/j.jembe.2011.02.021.

[ece373158-bib-0103] Wotherspoon, S. J. , M. D. Sumner , and S. Lisovksi . 2013. R Package SGAT: Solar/Satellite Geolocation for Animal Tracking. GitHub repository. https://github.com/SWotherspoon/SGAT.

[ece373158-bib-0104] Xavier, J. , and Y. Cherel . 2009. Cephalopd Beak Guide for the Southern Ocean. British Antarctic Survey.

[ece373158-bib-0105] Zuur, A. F. , E. N. Leno , and C. S. Elphick . 2010. “A Protocol for Data Exploration to Avoid Common Statistical Problems.” Methods in Ecology and Evolution 1, no. 1: 3–14. 10.1111/j.2041-210X.2009.00001.x.

